# 4-1BB Agonism as a strategy to license immune checkpoint blockade in glioblastoma

**DOI:** 10.18632/oncoscience.505

**Published:** 2020-05-14

**Authors:** Karolina Woroniecka, Peter E. Fecci

**Affiliations:** ^1^Preston Robert Tisch Brain Tumor Center, Duke University Medical Center, Durham, NC, USA; ^2^Department of Pathology, Duke University Medical Center, Durham, NC, USA; ^3^Department of Neurosurgery, Duke University Medical Center, Durham, NC, USA

**Keywords:** 4-1BB agonism, checkpoint blockade, glioblastoma, intracranial metastasis

Immune checkpoint blockade targeting PD-1 and CTLA-4 is an FDA-approved strategy in a number of solid tumors, yet has demonstrated limited success as a monotherapy in glioblastoma (GBM) [4]. GBM evades immunotherapies such as immune checkpoint blockade through inducing rampant T-cell dysfunction, including severe T cell exhaustion [5]. T cell exhaustion, characterized by the upregulation of multiple immune checkpoints, is a state of hyporesponsiveness to T cell stimulation. Strategies to sustain T cell activation within the CNS thus may improve the efficacy of immune checkpoint blockade strategies.

We recently uncovered that 4-1BB agonism may serve as an effective T cell activation strategy that licenses subsequent immune checkpoint blockade in GBM [6]. 4-1BB is a costimulatory receptor which accumulates on T cells upon activation, with subsequent downstream signaling powerfully augmenting T cell activation. 4-1BB has been targeted with agonist antibodies in clinical trials, yet has previously yielded only modest benefits in patients with solid tumors while also conferring risks for toxicity. Recently, studies have demonstrated substantial synergy of 4-1BB agonism in combination with immune checkpoint blockade, with potentially decreased risks of toxicity associated with either treatment alone [1]. This work has spurred interest in developing newer therapeutic tactics that offer the ability to limit off-target toxicity [3]. These promising data led us to investigate 4-1BB agonism as a strategy to license immune checkpoint blockade in GBM, which had not been previously explored in the CNS.

We initially examined 4-1BB expression on T-cells infiltrating human GBM tumors (TIL). Among TIL, 4-1BB expression was associated with an activated, “single positive” (PD-1+) phenotype rather than the exhausted, “triple positive” (PD-1+, TIM-3+, LAG-3+) phenotype. Accordingly, 4-1BB+ “single positive” TIL were significantly more functional as measured by IFN-γ production, than “triple positive” TIL. These findings suggested that 4-1BB may serve as a marker for non-exhausted TIL that may subsequently respond to immune checkpoint blockade. This may be of importance due to variable 4-1BB expression between patient samples – while many human GBM patient TIL expressed high levels of 4-1BB, there remained several samples which lacked 4-1BB. In the future, ascertaining whether TIL in a particular patient’s tumor express 4-1BB prior to initiating a potentially toxic therapy may be of value. 4-1BB expression furthermore appeared to correlate with functional response to 4-1BB stimulation when tested with an in vitro stimulation assay using a 4-1BB agonist antibody, suggesting that degree of 4-1BB expression may be a relevant functional predictor of therapeutic response.

These findings in human GBM TIL were recapitulated in murine models, allowing further evaluation of preclinical efficacy of this immunotherapeutic strategy. In a murine model of GBM, CT2A, we found that 4-1BB agonism and PD-1 blockade averted T cell exhaustion. Correspondingly, 4-1BB agonism significantly licensed PD-1 blockade in this preclinical model when used as a therapeutic strategy. Mice treated with PD-1 blockade alone did not demonstrate a survival benefit; mice treated with 4-1BB agonism had mildly prolonged median survival; while the combination of PD-1 blockade and 4-1BB agonism achieved 50% long-term survival. This immunotherapeutic strategy was found to be CD8+ T cell dependent. These encouraging findings demonstrate that the use of a T cell activating strategy such as 4-1BB agonism may provide the stimulus needed for the efficacy of subsequent immune checkpoint blockade.

We next sought to evaluate whether this strategy may be effective in metastatic brain tumors, such as lung cancer, melanoma, and breast cancer. Interestingly, we found that the combination of 4-1BB agonism and PD-1 blockade was most efficacious in our CT2A glioma model. We found that CD8+ T cells infiltrating CT2A were more likely to express 4-1BB than CD8+ T cells infiltrating other models, in a manner that appeared to correlate with the efficacy of combination treatment against that tumor. Similar findings were seen in tumors implanted peripherally. These findings imply that 4-1BB expression on the surface of CD8+ TIL may serve as an indicator of which tumor types, and potentially which patients, may respond to 4-1BB agonism. Whether or not patients with 4-1BB+ TIL may benefit from 4-1BB agonism as an adjunct to PD-1 blockade remains to be evaluated.

We lastly demonstrated that enhancing 4-1BB levels of CD8+ T cells through forced 4-1BB overexpression is sufficient to proffer an effective response to 4-1BB agonism and PD-1 blockade. These findings suggest that if TIL can be provided with 4-1BB expression, then 4-1BB agonism may be utilized as an adjunct to PD-1 blockade, even in those tumor types or patients whose TIL may lack native 4-1BB expression. While strategies such as TIL isolation, genetic manipulation, and subsequent adoptive transfer are certainly possibilities, these strategies are hampered by difficulty of processing, cost, and limited to each individual patient. Further studies to assess drivers of 4-1BB expression on TIL are needed, and may yield novel approaches to increase TIL 4-1BB expression.

Taken together, these findings suggest that 4-1BB agonism may be a viable immunotherapeutic treatment strategy to overcome resistance to PD-1 blockade in tumor types where TIL express high levels of 4-1BB such as GBM. Future studies will need to determine mechanistic drivers of 4-1BB expression amongst TIL across different tumor types and between patients, and to develop effective yet minimally toxic immunotherapeutic combinatorial strategies.
